# Research on Self-Balancing System of Autonomous Vehicles Based on Queuing Theory

**DOI:** 10.3390/s21134619

**Published:** 2021-07-05

**Authors:** Huanping Li, Jian Wang, Guopeng Bai, Xiaowei Hu

**Affiliations:** 1 School of Transportation Science and Engineering, Harbin Institute of Technology, Harbin 150090, China; wang_jian@hit.edu.cn (J.W.); xiaowei_hu@hit.edu.cn (X.H.); 2 Department of Civil and Environmental Engineering, University of Macau, Macau 999078, China; yc07450@umac.mo

**Keywords:** autonomous vehicle, self-balancing, queuing theory, MVA, energy

## Abstract

In order to explore the changes that autonomous vehicles on the road would bring to the current traffic and make full use of the intelligent features of autonomous vehicles, the article defines a self-balancing system of autonomous vehicles. Based on queuing theory and stochastic process, the self-balancing system model with self-balancing characteristics is established to balance the utilization rate of autonomous vehicles under the conditions of ensuring demand and avoiding an uneven distribution of vehicle resources in the road network. The performance indicators of the system are calculated by the MVA (Mean Value Analysis) method. The analysis results show that the self-balancing process could reduce the average waiting time of customers significantly in the system, alleviate the service pressure while ensuring travel demand, fundamentally solve the phenomenon of concentrated idleness after the use of vehicles in the current traffic, maximize the use of the mobile vehicles in the system, and realize the self-balancing of the traffic network while reducing environmental pollution and saving energy.

## 1. Introduction

Autonomous vehicles have the nature of shared bicycles and could also decrease in one place and increase in another place in an urban road network, causing an imbalance in the allocation of system resources. The most common direct solution for shared bicycles is to use a transport vehicle to evacuate them from the centralized area to facilitate the balanced use of the system. However, in the self-balancing system of fully autonomous vehicles set in this article, the above phenomenon would not occur. Instead, the autonomous vehicle could self-balance the centralized idle phenomenon of vehicles after use, perform self-balancing according to the traffic demand in the road network, and utilize the number of moving vehicles in the system to achieve self-balancing of the transportation network. The autonomous vehicle’s self-balance system could also ensure the service level of an urban network. In this paper, the fully automatic vehicle transportation system could not only avoid the parking difficulty of customers after they reaching destination but also reduce the number of urban parking lots, especially in the prosperous areas of cities. For individuals, it could save time and money. After improving the traffic environment, urban traffic customers do not have to purchase a private vehicle or worry about the parking spaces and traffic congestion. Even urban air pollution could be effectively solved in the fully automatic driving traffic system, which could improve the travel efficiency of the city, save the traveler and the whole city travel cost, and alleviate the loss of natural energy caused by urban operation. Travelers only need to buy their own traffic service.

The purpose of this article is to establish a self-balancing model for autonomous driving in the road network and to evaluate the potential benefits of the self-balancing road network system for autonomous vehicles. In 2012, Pavone et al. [1] regarded travelers and vehicles of the transportation system as continuous and studied the mobile travel demand algorithm with mobility through the method of fluid approximation, but to some extent, they ignored the random fluctuations in the system and did not consider the calculation of key performance indicators such as the availability of station and the waiting time of users. Shoup [2] (2005) conducted a study on 22 sample data in the United States, and the results showed that in urban traffic operations, about 30% of drivers are looking for parking spaces during urban traffic operations. At the same time, the average daily usage of private cars is less than two hours, but they occupy parking spaces during idle time. To solve these problems, some scholars consider the best site selection and site capacity in the transportation system from a strategic level. To a large extent, the existing problems in the existing transportation system have been alleviated by their studies. Some scholars alleviate the current traffic congestion problem by assigning an appropriate number of vehicles at some stations while ensuring travel demand and reducing the number of vehicles in the transportation system as much as possible. For example, George and Xia (2011) [3] studied the size of the fleet that should be maintained if there are unlimited parking spaces in the system when the travel demand remains the same. They also studied the situation of fleet rental in the closed-loop queuing network and optimized the availability of rental vehicles at the road network nodes as well as service indicators of rental vehicles. Although this model is aimed at the vehicle rental system, it views the queuing rental system from the perspective of vehicles, determining the size and profit of the fleet and the sensitivity of various cost scenarios, which can provide strategic decisions for the operation system. Some scholars improve the efficiency of the system by optimizing pickup and delivery problems [4]. Shu et al. (2010) [5] established a network flow model based on stochastic processes and forecasted demand based on public transportation in Singapore to verify these conclusions. Lin and Yang (2011) [6] expressed similar problems with mathematical models in his paper. Fricker and Gast (2012) and Fricker et al. (2012) [7,8] considered the optimal vehicle size in a particular city, assuming that the traffic demand at different times in the city is a fixed value and all traffic station nodes have the same capacity *k*, and the results show that if there is no operational system management strategy, even if the optimal fleet size is formulated, there is a probability 2/(k + 2) that any given station is empty or full. The Apollo system in 2011 and the Car2go system in 2008 are a big step toward fully autonomous vehicles [9,10]. Bullo et al. (2011) and Pavone et al. (2011) [11] established a vehicle routing selection model: namely, the DPDP (Dynamic Pickup and Delivery Problem in the Vehicle Dynamic Pickup and Delivery Network). The articles of George and Xia (2011) and Washerhole and Jost (2013) [12] also sought to solve related problems by modeling a traffic network containing traditional (human-driven) shared vehicles within the framework of the Jackson network (Serfozo, 1999) [13], but it does not consider the self-balancing problem of autonomous vehicles. A static version of one balancing problem has been analyzed by Raviv et al. (2013) [14] and a dynamic one has been analyzed by Contardo et al. (2012) [15]. Dong and Song (2009) [16] studied the repositioning and fleet-sizing problem for liner shipping systems, developing a solution procedure using simulation and evolutionary algorithms. Sayarshad and Marler (2010) [17] and Sayarshad et al. (2010) [18] studied the use of multi-objective optimization formulations for decision making in the railway industry. If the system is a self-driving and self-balancing transportation system, it can fundamentally solve the waste of resource allocation and traffic congestion. On the basis of reducing the empty driving rate of vehicles (similar to taxis), it can also control vehicles to stop at any time. When there is a travel demand at the city’s road network nodes, the system could select the preferred choice of autonomous vehicles to serve the travel needs of the road network, and even urban air pollution caused by vehicle exhaust could be effectively improved.

The article is mainly divided into four parts: The second part mainly describes the mathematical model that the article should use; the third part is the subject content of the article, that is, the self-balancing system of autonomous driving; the fourth part is a small case analysis to verify the effectiveness of the model; the fifth part is a summary and prospect.

Specifically, the article mainly has the following contributions:

(1) Define a traffic system with self-balancing of autonomous vehicles;
(2) Based on the queuing theory, a road network model with self-balancing characteristics is established;
(3) The balance strategy reduces the waiting time of the system;
(4) While increasing service efficiency, the service intensity of the system is reduced;
(5) Improve the operation efficiency of the transportation system.

Autonomous driving serves travelers based on the actual travel needs in the system and avoids the empty driving state without target. It still serves the travel demand before autonomous driving occurs, so it would not increase the travel burden. The autonomous vehicles self-balancing system could fundamentally reduce the number of vehicles in the road system and at the same time solve the problem of difficult parking. Due to the interconnection phenomenon of vehicles in fully autonomous vehicles, congestion during peak hours will also be weakened.

Therefore, the self-balancing system of automatic driving is a key link to improve the urban travel service and is also an indispensable support part for autonomous vehicles to serve urban customers. It provides foundational research of the development of intelligent transportation systems, the construction of a harmonious traffic environment, and the development of e-commerce.

## 2. Modeling Basis and Research Methods

In order to explain the network characteristics of the self-balancing system when the road network is full of autonomous driving vehicles, this paper projects the defined self-balancing traffic system of autonomous vehicles into the Jackson closed-loop network and uses the queuing theory to model and analyze the system. For a regional network, the total number of trips in the road network does not change much over time. That is, there is no external input in the system [19], the total number of trips in the system is a constant *K*, and all travelers circulate. Therefore, in this travel mode, the self-balancing characteristic of the autonomous driving vehicle between different stations in the system is particularly important, so as to meet the travel needs of the system under the condition of minimizing the autonomous driving vehicle. Since vehicle sharing cannot guarantee the flexibility of individual needs, the phenomenon of sharing rides is not considered in the automatic driving self-balancing system in this paper. However, compared with the resource waste caused by private cars, which are idle for about 95% of the time in a life cycle but occupy a large amount of parking lots, self-balancing autonomous vehicles are the most efficient use of mobility and mobility for both vehicles and individuals. The purpose of this paper is to reflect the benefits that could be brought to the current traffic system when the intelligent characteristics of the autonomous vehicles are fully utilized. We attempt to show the changes before and after the self-balancing of the autonomous vehicles from the perspective of passengers and the system. For passengers, the smaller the waiting time and queue length, the better. For the system, the average waiting time of passengers, the average queue at the system node, and the service intensity of the system are all considered on the basis of ensuring the passenger travel demand. Queuing theory is a very mature theory that just could fully reflect these system performances.

### 2.1. Queuing Network of Autonomous Vehicles

In this paper, the autonomous vehicles are put into the closed-loop Jackson network, and the knowledge of queuing theory is used to determine how the autonomous vehicles of the system can achieve the self-balancing state while better ensuring the demand of the road network.

Suppose there are *M* nodes in the network, and satisfy:

(1) The service rate of node *i* is related to its queue length. When there are *n_i_* customers in the queue of node *i*, the service rate is $u_{i}\left(n\right)$, and the service time of each node is independent and follows a negative exponential distribution;
(2) Node *i* receives Poisson flow with a rate of $\lambda_{i}$;
(3) Passengers transfer to node *j* with probability $p_{ij}$ after completion of service at node *i*, and the transfer probability has Markov characteristics;
(4) The network is closed, with a fixed number of passengers *K*.

Since the network is closed, the external input $v_{i}=0\;\sum_{j=1}^{m}p_{ij}=1,i=\left\{1,2,\ldots M\right\}$ and *K* is fixed, the state space of the network Φ is limited, that is:

(1) $$\Phi =\{\left(n_{1},n_{2},\cdots ,n_{M}\right)|n_{i}\geq \;0,\sum_{i=1}^{M}n_{i}=K\}.$$

The cardinal number of Φ is:

$$\left|\Phi \right|=\left(\begin{array}{l} K+M-1\\ M-1 \end{array}\right).$$

As Φ  is limited, there is always a stable state in the case of no external arrival and departure, and the routing probability $p_{ij}$ satisfies the requirement:

$$\sum_{j=1}^{M}p_{ij}=1,\;i=1,2,\cdots ,M.$$

The flow equation at the node *i* satisfies:

(2) $$\lambda_{i}=\sum_{j=1}^{M}\lambda_{ji}p_{ji}\;i=1,2,\cdots ,M.$$

The routing probability matrix is defined as ***R***: $R=(p_{ij}|i,j=1,\cdots ,M)$; then, it can be known from the linear algebra homogeneous linear equation definition that Equation (2) satisfies: λ(I−R)=0; ***I*** is the identity matrix, $\lambda =\left\{\lambda_{i}\right\}$. As |I−R|=0, $\lambda =\left\{\lambda_{i}\right\}$ has an infinite number of solutions. The different solutions differ by a multiplier factor. Let $\left(e_{1},e_{2},\cdots ,e_{M}\right)$ be any set of non-zero solutions; then, $e_{i}$ is proportional to $\lambda_{i}$: that is, $e_{i}=\varepsilon \lambda_{i}$ and ε is constant. In general, the element of $\left(e_{1},e_{2},\cdots ,e_{M}\right)$ could be fixed as a convenient value, such as $e_{1}=1$; that is, once there is access to node 1, the average access to node *i* is $e_{i}$ times, so $e_{i}$ is also called the relative access rate of node *i*. We can also form a probability distribution $\sum_{i=1}^{M}e_{i}=1$ by normalizing $\left(e_{1},e_{2},\cdots ,e_{M}\right)$; thus, a network with only one customer could be considered to walk randomly in a node set. The probability of $e_{i}$ customer at node *i* is MC in state *i*. If $\left(e_{1},e_{2},\cdots ,e_{M}\right)$ satisfies Equation (2), we do not need to know the specific value. The definition of $\sigma_{i}=e_{i}/u_{i}$, which is proportional to the service intensity $\rho_{i}=\lambda_{i}/u_{i}$ of node *i*.

### 2.2. Probability Distribution in Steady State

Assuming that the queue at node *i* is *j* and the service rate is $u_{i}(j)$, the joint probability distribution under the stable state is:

(3) $$P(n_{1},n_{2},\cdots ,n_{M})=\frac{1}{G}\prod_{i=1}^{M}\sigma_{i}(n_{i})$$

(4) $$\sigma_{i}(n_{i})=\left(\frac{e_{i}^{n_{i}}}{\prod_{j=1}^{n_{i}}u_{i}\left(j\right)}\right),\sum_{i=1}^{M}n_{i}=K.$$

In order to make $P(n_{1},n_{2},\cdots ,n_{M})$ probability quantifiable, the normalized constant *G* is defined:

(5) $$G=\sum_{n\in \Phi }\prod_{i=1}^{M}\sigma_{i}(n_{i}).$$

Let $e_{i}^{*}=\varepsilon e_{i}(i=1,2,\cdots ,M)$, sum over all states: $G^{*}=G\varepsilon^{K}$. The corresponding joint distribution probability $P^{*}(n)$ is:

(6) $$P^{*}(n)=\frac{1}{G^{*}}GP(n)\varepsilon^{\sum n_{i}}.$$

By combining the above two expressions, we can get: $P^{*}(n)=P(n)$, that is, $\left\{\lambda_{i}\right\}$ is arbitrary and affected by a multiplicative factor.

### 2.3. State Parameter Determination

Let $n_{i}=0$ and sum over the joint probability distribution; then, the probability of node *M* being idle could be obtained as follows:

$$P(n_{i}=0)=\frac{1}{G(M,K)}\sum_{\begin{array}{l} n\in \Phi \\ n_{i}=0 \end{array}}\prod_{i=1}^{M-1}\sigma_{i}^{n_{i}}=\frac{G(M-1,K)}{G(M,K)}.$$

(1) Queue length of node *i*:
  $$\begin{array}{ll} P(n_{i}\geq k) & =\frac{1}{G(M,K)}\sum_{\begin{array}{l} n\in \Phi (M,K)\\ n_{i}\geq k \end{array}}\prod_{j=1}^{M}\sigma_{j}^{n_{i}}\\ & =\frac{\sigma_{i}^{k}}{G(M,K)}\sum_{\begin{array}{l} m_{j}=n_{j}(i\neq j)\\ m_{i}=n_{i}-k\\ n\in \Phi (M,K)\\ n_{i}\geq k \end{array}}\prod_{j=1}^{M}\sigma_{j}^{m_{j}}. \end{array}$$

The summation part of the above equation is m∈(Φ,K−k), so the probability of $P(N_{i}\geq k)$ is:

(7) $$P(n_{i}\geq k)=\sigma_{i}^{k}\frac{G(M,K-k)}{G(M,K)}\;i=1,2,\cdots ,M.$$

The queue length distribution of node *i* is:

(8) $$\begin{array}{ll} Q_{i}(k) & =P(n_{i}\geq k)-P(n_{i}\geq k+1)\\ & =\sigma_{i}^{k}\left(\frac{G(M,K-k)-\sigma_{i}(M,K-k-1)}{G(M,K)}\right) \end{array}.$$

The service intensity of node *i* is:

(9) $$\rho_{i}=\sigma_{i}\frac{G(M,K-k)}{G(M,K)}.$$

Then the actual throughput of node *i* is:

(10) $$\phi_{i}=\mu_{i}\rho_{i}=e_{i}\frac{G(M,K-k)}{G(M,K)}.$$

Let $n_{i}=1$ and define the relative utilization of node *i* as:

(11) $$\delta_{i}=\sigma_{i}(n_{i}=1)=\frac{e_{i}}{u_{i}(1)}.$$

Then, Equation (8) could be expressed as:

(12) $$\begin{array}{ll} Q_{i}(k) & =P(n_{i}\geq k)-P(n_{i}\geq k+1)\\ & =\delta_{i}^{k}\left(\frac{G(M,K-k)-\delta_{i}(M,K-k-1)}{G(M,K)}\right) \end{array}.$$

(2) The probability of node *i* being idle is:
  $$\begin{array}{l} Q_{i}(k=0)=P(n_{i}\geq k)-P(n_{i}\geq k+1)\\ =\left(\frac{G(M,K)-\delta_{i}(M,K-1)}{G(M,K)}\right) \end{array}.$$

The utilization rate (service intensity) of node *i* is:

(13) $$\rho_{i}=\frac{\delta_{i}(M,K-1)}{G(M,K)}.$$

Then, the throughput of node *i* is:

(14) $$\phi_{i}=\mu_{i}\rho_{i}=e_{i}\frac{G(M,K-1)}{G(M,K)}.$$

(3) The average queue length of node *i*:

$q_{i}\left(K\right)$ represents the average queue length of node *i* in a network with *K* customers in a stable state and could be obtained from the cumulative probability triangle sum:

$$\begin{array}{ll} q_{i}(K) & =\sum_{j=1}^{K}jP(n_{i}=j)\\ & =\sum_{j=1}^{K}\sum_{j=k}^{K}P(n_{i}=j)\\ & =\sum_{k=1}^{K}P(n_{i}\geq k) \end{array}.$$

From Equation (8), the average queue length of node *i* is:

(15) $$q_{i}(K)=\frac{1}{G(M,K)}\sum_{k=1}^{K}\sigma_{i}^{k}G(M,K-k)\;i=1,2,\cdots ,M.$$

From Equation (11), we can get:

(16) $$q_{i}(K)=\frac{1}{G(M,K)}\sum_{k=1}^{K}\delta_{i}^{k}G(M,K-k)\;i=1,2,\cdots ,M.$$

## 3. Self-Balancing System for Fully Autonomous Vehicles

Autonomous vehicles could operate without drivers. In an urban environment, autonomous vehicles can transport passengers on their own. However, autonomous vehicles have the nature of shared bicycles and would also decrease in one place and increase in another place in an urban road network, causing an imbalance in the allocation of system resources. The most common direct solution for shared bicycles is to use a transport vehicle to evacuate them from the centralized area to facilitate the balanced use of the system.

However, in the self-balancing system of fully autonomous vehicles set in this article, the above phenomenon will not occur. Instead, the autonomous vehicle could self-balance the centralized idle phenomenon of vehicles after use, perform self-balancing according to the traffic demand in the road network, and utilize the number of moving vehicles in the system to achieve self-balancing of the transportation network. So, it is important to outline this self-balancing mode and determine how to achieve self-balancing so that the urban network could achieve a self-balancing state while ensuring the traffic demand, optimizing the control cost output, reducing the waiting time of the system, and improving the operation efficiency of the system. Based on these problems, this paper puts the autonomous vehicles into the closed-loop Jackson network and uses the knowledge of queuing theory to determine how the autonomous vehicles in the system could better achieve a self-balancing state while ensuring the demand of the road network. Since car sharing cannot guarantee the flexibility of individual needs, the autonomous vehicles self-balancing system in this article does not consider the phenomenon of ride-sharing, but compared with the private car—which is idle about 95% of the time in a life cycle, and at the same time, it needs to occupy a large number of parking lots, resulting in a waste of resources—self-balancing autonomous vehicles are the most efficient use of vehicles and mobility for both vehicles and individuals.

### 3.1. Self-Balancing System Framework

**Figure 1:**
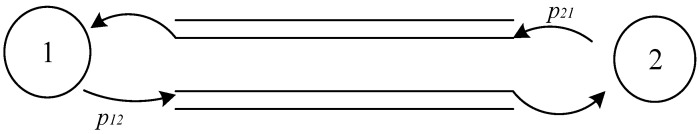
Traffic network diagram of two stations.

This paper uses queuing theory as the basic framework to establish a self-balancing model for autonomous vehicles. Assume that in a given road network, there are *M* stations and *H* autonomous vehicles providing services for customers to travel. Customer arrival obeys the Poisson distribution, and the rate of arrival at node *i* is $\lambda_{i}$. When the customer arrives at *i*, the destination *j* is selected according to the routing probability $p_{ij}$, where $\sum_{j}p_{ij}=1$. According to the above hypothesis, the routing probability ${\left\{p_{ij}\right\}}_{ij}$ is an irreducible Markov chain. If there is an autonomous vehicle parked at node *i*, the customer can take the autonomous vehicle to the selected destination. If there is no automatic vehicle available for service at node *i*, customers will leave the queuing system immediately; that is, this system is a queuing system with customer loss. It is assumed that there are enough parking spaces for autonomous vehicles at each station so that vehicles could stop immediately when arriving at the station. Autonomous vehicles can travel automatically in the network, predicting and participating in the future traffic demand with the final state of each end of service. The following Figure 1 shows a closed-loop queueing network with two nodes.

### 3.2. Queue Network Model of Self-Balancing System

The key to deploying the autonomous vehicles self-balancing system to the Jackson network is to consider an abstract queuing network. *SN* represents the single-server (SS) station node in the network; *IN* represents the infinite server road nodes. In a queuing network without automatic driving or automatic driving self-balancing, the vehicles cannot balance after completing the travel service. In this case, the vehicle will form a queuing system at the station node to wait for customers to arrive. After the customer arrives, they accept the vehicle service; then, the vehicle leaves the *SN* node to the *IN* node and completes the OD itinerary selected by the customer. This process is that the vehicle leaves the station node *i*, moves to the road node *ij* with the routing probability $p_{ij}$, and then moves to the station node *j* with the probability 1. Based on the above definition, a closed-loop queuing network system is established. Since in a fully automated driving road traffic system, as long as the road planning is reasonable, the automatic driving vehicles could be changed in the form of a fleet. There is almost no congestion state, only the level of service exists, so the road node of this model is an *IS* queue; that is, there is no blocking effect. Use set *S* to represent the *SN* nodes of the fully automated driving road network and set *I* to represent the *IN* road nodes. Each station is mapped into an *SN* node, and each road is mapped into an *IN* node. Θ represents the set of all nodes in the abstract queuing system; that is, Θ=S ∪ I. Since each *SN* node is connected to another *IN* node, and $p_{ii}=0$, the number of nodes in the road network is $\Theta (\Theta -1)+\Theta =\Theta^{2}$; that is, the cardinality of the set Θ is $\left|\Theta \right|=\Theta^{2}$. For each *IN* node *I* ∈ *I*, O(*i*) and D(*i*) are the departure and arrival node of *i*, respectively. As mentioned above, in the abstract queuing network set, vehicles move between the *SN* node and *IN* node according to routing matrix ${\left\{p_{ij}\right\}}_{ij}$.

(17) $$p_{ij}=\left\{ \begin{array}{ll} p_{il} & i\in S,j\in I;\;i=O(j),\;l=D(j)\\ 1 & i\in I,j\in S;\\ 0 & otherwise \end{array}\right.$$

The service time of any i∈Θ is distributed exponentially, and the service rate is as follows:

(18) $$u_{i}(h)=\left\{ \begin{array}{ll} \lambda_{i} & i\in S\\ h\cdot u_{jf} & i\in I,j\in O(i),f\in D(i) \end{array}\right.$$

where *h* ∈{0,1,…H} is the number of autonomous vehicles at node *i*, $u_{jf}=1/T_{jf}$.

The autonomous vehicles in the model balance themselves in the road network according to real-time traffic demand. While maintaining the Jackson queuing network framework, this paper focuses on the stochastic equilibrium strategy of autonomous driving in the transportation system. Assume that the autonomous vehicle balances itself through potential travel demand (virtual passengers) at each station node *i*. The potential travel is Poisson arrival with an arrival rate of $\psi_{i}$ and the potential travel demand is independent of the actual demand that has been generated at the moment. The autonomous vehicle serves the potential trip to node *j* according to the probability $\theta_{ij}\left(\sum_{j}\theta_{ij}=1,\theta_{ii}=0\right)$. When the potential travel demand is generated, if the station is empty, there will be customer loss just as in the real travel demand. The model encourages autonomous vehicles to self-balance according to traffic conditions but does not force autonomous vehicles to self-balance.

When the autonomous vehicle considers self-balance according to the current traffic conditions, it will combine the actual travel demand with the potential travel demand. In essence, both of them are the travel demand to be served by the autonomous vehicle. Therefore, when the potential travel demand is generated, the mathematical structure of the model will not be changed. All travel demands are generated in set Θ. $\left\{{Ε}_{t}^{\left(i\right)},t>o\right\}$ is the total arrival amount of real and virtual passengers at the station node *i* ∈ *S*, and the arrival rate is ${\dddot{\lambda }}_{i}$. Since the Poisson process $E_{i}^{\left(t\right)}$ is a superposition of two independent Poisson processes, ${\dddot{\lambda }}_{i}$ is as follows:

$${\dddot{\lambda }}_{i}=\lambda_{i}+\psi_{i}.$$

The self-balancing process of automatic driving could be seen as the Bernoulli split result of the total demand in the case of the potential demand ratio is $\tau_{i}$:

$$\psi_{i}=\tau_{i}{\dddot{\lambda }}_{i}$$

$$\lambda_{i}=(1-\tau_{i}){\dddot{\lambda }}_{i}.$$

The probability that a travel demand arrives at station node *i* and chooses *j* as the destination in $\left\{{Ε}_{t}^{\left(i\right)},t>o\right\}$ is ${\dddot{p}}_{ij}$:

$${\dddot{p}}_{ij}={\overset{⌢}{p}}_{ij}\tau_{i}+p_{ij}(1-\tau_{i})=\theta_{ij}\tau_{i}+p_{ij}(1-\tau_{i}).$$

The probability of potential travel demand choosing station node *j* as its destination is ${\hat{p}}_{ij}$. The true demand chooses station node *j* as its destination probability is $p_{ij}$. The self-balancing process of the autonomous vehicle is defined according to the routing matrix in Equation (17) and the given service rate in Equation (18).

The routing probability matrix and service rate with self-balancing process are:

(19) $${\dddot{p}}_{ij}=\left\{ \begin{array}{ll} {\dddot{p}}_{il} & i\in S,j\in I;\;i=O(j),l=D(j)\\ 1 & i\in I,\;j\in S;\;\\ 0 & otherwise \end{array}\right..$$

The service time of any i∈Θ is distributed exponentially, and the service rate is as follows:

(20) $$u_{i}(h)=\left\{ \begin{array}{ll} {\dddot{\lambda }}_{i} & \;i\in S\\ h\cdot u_{jf} & i\in I,\;j\in O(i),f\in D(i) \end{array}\right..$$

The node *M* in the queuing network described in the second part of the article refers to *M* station nodes, such as $\delta_{i}$ corresponds to the *i*th *SN* node.

### 3.3. Establishment of Self-Balancing Optimization Model

In the self-balancing process of the autonomous vehicle, $\delta_{S}^{\max}=\max_{i\in S}\delta_{i}$ is defined as the maximum relative utilization of station node *SN*. When the number of autonomous vehicles *H* tends to *+∞*, the availability of set $B=\{i\in S,\delta_{i}=\delta_{S}^{\max}\}$ is infinitely close to 1. No matter what *H* is, the availability of other sites is strictly less than 1; that is, if the system’s autonomous vehicles do not self-balance, no matter how many autonomous vehicles the station keeps, there is a loss of customers in the system. For set *B*, there are the following results [4].

If $\delta_{i}\geq \delta_{j}$, then $\rho_{i}(H)\geq \rho_{j}(H)\;\forall i,j\in S,\;H\in N^{+}$. Then, there are:

(21) $$\begin{array}{l} \underset{H\to \infty }{\lim}\rho_{i}(H)=\delta_{i}/\delta_{i}^{\max}\;\forall i\in S\\ \underset{H\to \infty }{\lim}L_{i}(H)=\delta_{i}/\delta_{i}^{\max}\;\forall i\in I \end{array}.$$

*L_i_* is a function of *H*, which is the expected queue length in a steady state. Muntz and Wong confirmed the asymptotic behavior of Equation (21).

In this paper, the self-balancing model of fully autonomous driving vehicles is proposed, which is expected to (1) achieve the equilibrium state of the road network; (2) provide a fair service opportunity for the travel demand of the road network; (3) improve the service efficiency of the road network; and (4) reduce the average customers waiting time of the road network. The goal of self-balancing is to control $\psi_{i}$ and $\theta_{ij}$. The purpose of the model is to achieve a balanced allocation of vehicle resources on the basis of meeting the demand and improving the operating efficiency of the system. In this process, all $\delta_{i}$ in *S* are equal. The mean value of the self-balancing rate between station nodes *i* and *j* is $\theta_{ij}\psi_{j}$. Since there is no ride-sharing phenomenon in this article, one customer corresponds to one autonomous vehicle, and there are enough autonomous vehicles parked at the station node; that is, this article mainly focuses on how many autonomous vehicles are needed for self-balancing during the self-balancing process rather than how many autonomous vehicles should be stored in the system.

In the self-balancing Jackson queuing model of autonomous driving in this paper, the objective function is:

$$\underset{\psi_{i},\theta_{ij}}{\min}\;\sum_{i,j}\theta_{ij}\psi_{j}$$

(22) $$\begin{array}{ll} S.t & \;\lambda_{j}+\sum_{i}p_{ij}\lambda_{i}+\sum_{i}\theta_{ij}\psi_{i}\geq \psi_{j}\\ & \;\sum_{j}\theta_{ij}=1\\ & \;0\leq \theta_{ij}\leq 1,\psi_{i}\geq 0\;i,j\in \{1,\cdots ,M\} \end{array}.$$

### 3.4. Self-Balancing System Performance Index Calculation

According to the self-balancing closed-loop Jackson queuing network model established above for autonomous driving and Section 3.2, by solving the simplified station node flow equation, the relative throughput of the station node *SN* is obtained:

(23) $$e_{i}=\sum_{j\in S}e_{j}{\dddot{p}}_{ri}\;i\in S.$$

In the model established in this article, since the set station node *SN* is independent, the relative throughput of the road node *IS* is:

(24) $$e_{i}=e_{O(i)}{\dddot{p}}_{O(i)D(i)}\;i\in I.$$

The ${\left\{\psi_{i}\right\}}_{i}$ and ${\left\{\theta_{ij}\right\}}_{ij}$, i∈S generated by any autonomous vehicle rebalancing strategy in this paper satisfy:

$$\begin{array}{l} \;\delta_{i}>0\\ \;(\lambda_{i}+\psi_{i})\delta_{i}=\sum_{j\in S}\delta_{j}(\theta_{ji}\psi_{j}+p_{ji}\lambda_{j}) \end{array}.$$

According to Equation (23), the relative throughput $e_{i}$ of the road network nodes is calculated. In order to avoid the explicit calculation of the normalization constant G, a commonly used iterative algorithm is selected in this paper, Mean Value Analysis (MVA), to process *SN* and *IS* nodes, and the MVA algorithm can avoid the floating-point error inherent in the convolution algorithm. The MVA algorithm is a simple application based on the results of Little’s formula. Here, we only consider the average value and try to avoid directly dealing with the steady-state probability.

If the node i∈I, the average waiting time is the same as the average service time, so:

$$T_{wi}(k)=\frac{1}{\mu_{i}(1)}=T_{O(i)D(i)\;}i\in I.$$

If i∈S, the following recursive relationship could be obtained from the observer’s perspective, where *k* = 1, …, *K*, is the number of customers in the iterative algorithm:

$$T_{wi}(k)=\frac{1}{u_{i}}[q_{i}(k-1)+1]=\frac{1}{{\dddot{\lambda }}_{i}}[q_{i}(k-1)+1]\;i\in S.$$

The length of the queue in the closed-loop network is:

$$q_{i}(k)=e_{i}T_{wi}(k)\frac{k}{\sum_{i}^{\Theta }e_{i}T_{wi}(k)}=\frac{ke_{i}T_{wi}(k)}{\sum_{i}^{\Theta }e_{i}T_{wi}(k)}\;i\in \Theta .$$

According to the initial condition *T_wi_* (0) = *q_i_* (0) = 0, the values of *T_wi_* (1) and *q_i_* (1), 1 ≤ *i* ≤ *S* can be obtained; then, the value when *k* = 2 can be obtained, and so on, until the iteration reaches the expected value. In each iteration, 2*S* + 1 is calculated. In an urban transportation system serving customers, the average waiting time and queue length could measure the operation efficiency of an urban travel service system. For customers, the shorter the waiting time, the better and the shorter the queue length, and the better for a transportation system. For an operating system, the shorter the average waiting time, the better, and the smaller the operation intensity of the system nodes, the smaller the operation burden of the system would be. Therefore, this paper selects these commonly used measurement indicators in queuing theory and introduces the MVA method to simplify the calculation; then, it iteratively calculates the indicators by setting the initial value.

Finally, the flow/actual throughput of each station is given by the Little theorem [20] (Bertsekas et al., 1992, P. 152): $\phi_{i}(k)=q_{i}(k)/T_{wi}(k)\;,\;i\in S$. Combined with Equations (11), (13) and (14), the service intensity of each site after self-balancing can be easily obtained as follows: $\rho_{i}(k)=\phi_{i}(k)/{\dddot{\lambda }}_{i}$; if there is no self-balancing, it is: $\rho_{i}(k)=\phi_{i}(k)/\lambda_{i}$. This program could be well extended to a large number of stations and vehicles. An example analysis is carried out in Section 4 to evaluate the potential performance of the autonomous vehicles self-balancing system.

## 4. Experimental Analysis

**Figure 2:**
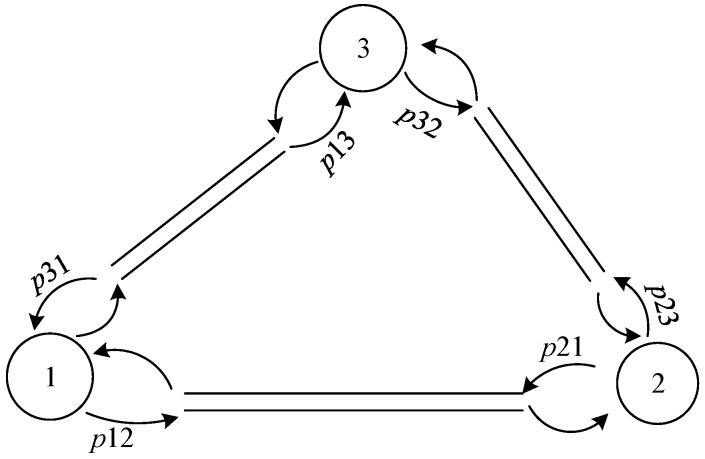
Traffic network diagram of three stations.

In order to verify the effectiveness of the model, the article considers a small road network with three sites, as shown in the following Figure 2.

Assuming that the arrival rate of each station node is λ=[0.3,0.4,0.3] and the routing distribution is $\;p_{ij}$, *i*,*j* ∈{1,2,3} customers form a first-come, first-serve queue at each station waiting for the service of autonomous vehicles. First, a route allocation matrix is given as:

$$p_{ij}=\left[\begin{array}{ccc} 0 & 0.3 & 0.7\\ 0.5 & 0 & 0.5\\ 0.6 & 0.4 & 0 \end{array}\right].$$

Randomly generate a potential travel demand ψ=[0.2,0.5,0.3] at nodes. Since the travel demand at the three nodes in the road network selected in this paper is balanced, the potential travel routing probability ***θ*** is

$$\theta =\left[\begin{array}{ccc} 0 & b & 1-b\\ a & 0 & 1-a\\ c & 1-c & 0 \end{array}\right].$$

### 4.1. Comparative Analysis before and after Self-Balancing

This section analyzes the changes of system performance indicators before and after self-balancing of autonomous vehicles in the closed-loop queuing network and makes a comparative analysis of these indicators.

When there is no autonomous vehicles self-balance phenomenon, according to the routing probability and flow equation, it could be known that the relative pass rate of nodes in the closed-loop queuing network meets:

$$\begin{array}{l} e_{1}=\frac{0.8}{0.85}e_{3}\\ e_{2}=\frac{0.58}{0.85}e_{3} \end{array}.$$

If *e*_3_ = 0.85, then *e*_1_ = 0.8 and *e*_2_ = 0.58.

**Figure 3:**
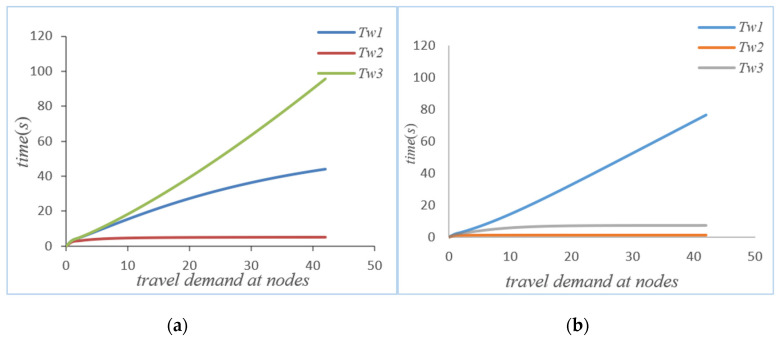
(**a**) Waiting time without self-balancing; (**b**) waiting time with self-balancing.

**Figure 4:**
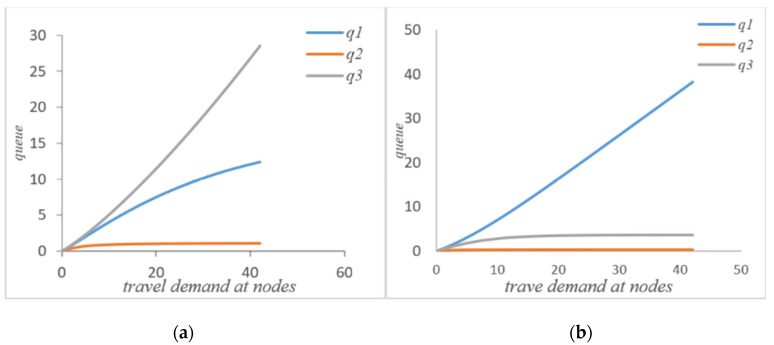
(**a**) Queue length without self-balancing; (**b**) queue length with self-balancing.

**Figure 5:**
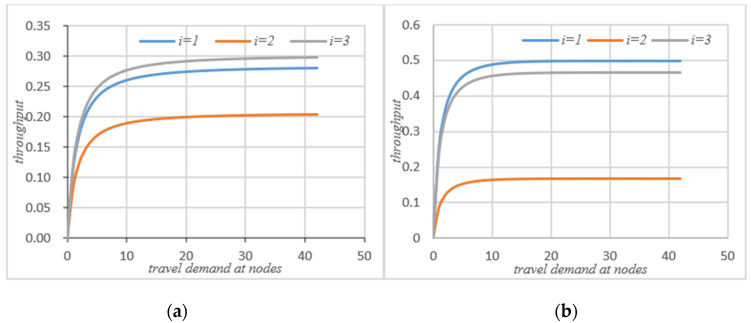
(**a**) Actual throughput without self-balance; (**b**) actual throughput with self-balance.

**Figure 6:**
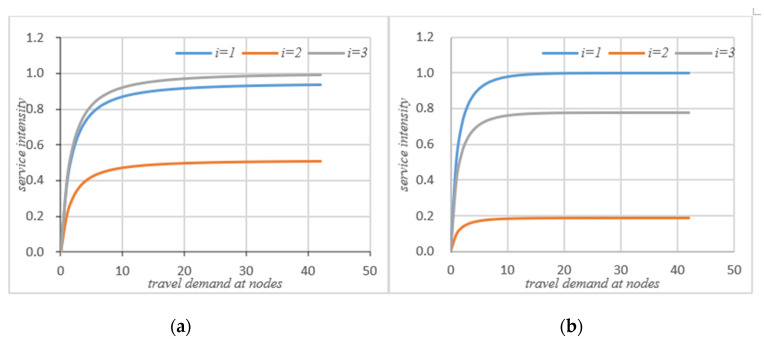
(**a**) Service intensity without self-balancing; (**b**)service intensity with self-balancing.

Initial conditions: $T_{wi}(0)=q_{i}(0)=0$,k={1,2,⋯K}, i = {1, 2, 3}; through the iteration of the MVA method, the performance indicators at the nodes in the road network without self-balancing of autonomous vehicles can be obtained, such as average waiting time, queue length, actual throughput, and the service intensity at nodes. When the system’s autonomous vehicles are self-balancing according to potential travel needs, based on *ψ* and routing probability ***θ*** and on the basis of the flow equation, the system equation can be iterated through the MVA method to obtain the average waiting time, the queue length, the actual throughput, and the service intensity at the node in the road network, as shown in Figure 3, Figure 4, Figure 5 and Figure 6 below.

**Figure 7:**
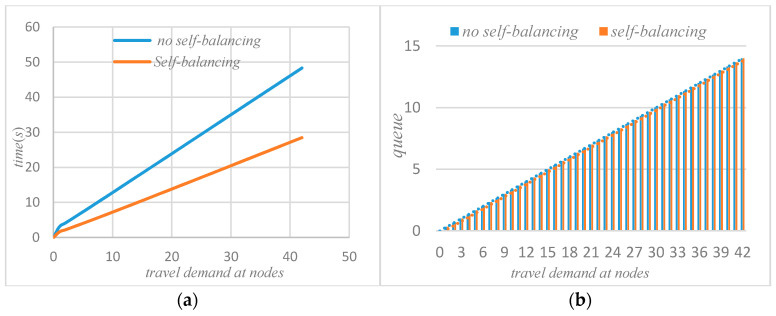
(**a**) Comparison of average waiting time; (**b**) Comparison of average queue.

As can be seen from Figure 3a,b, if the autonomous vehicle in the system adopts a self-balancing strategy, not every node average waiting time will be reduced. However, from the perspective of the system, it can be seen that the waiting time of node 1 has increased, but the waiting times of node 2 and node 3 are significantly reduced after the autonomous vehicle self-balancing. According to Figure 7, the average waiting time of the system has been significantly reduced after the self-balancing of the autonomous vehicle. Figure 4a,b compare the changes of queue length at nodes before and after the self-balancing of autonomous vehicles in the system under the same travel demand. The horizontal axis represents travel demand, and the vertical axis represents queue length.

It can be seen that after self-balancing, the queue length of node 1 has increased but the queue lengths of nodes 2 and 3 have been significantly reduced. Corresponding to the waiting time at the node in Figure 3, it can be seen that the self-balancing process is not a process of reducing the queue length at each node but rather a process of optimizing the allocation of system resources based on the coordination and balance process. Combined with Figure 7b, since the system is a closed-loop system, the number of customers remains unchanged, and the self-balancing process of autonomous driving is essentially a process of optimizing the use of resources, so the average queue length of the system will not change. Figure 5a,b compare the change of traffic volume at the nodes before and after the self-balancing of autonomous vehicles in the system under the same travel conditions. The horizontal axis represents the travel demand, and the vertical axis represents the actual passing capacity at the node.

**Figure 8:**
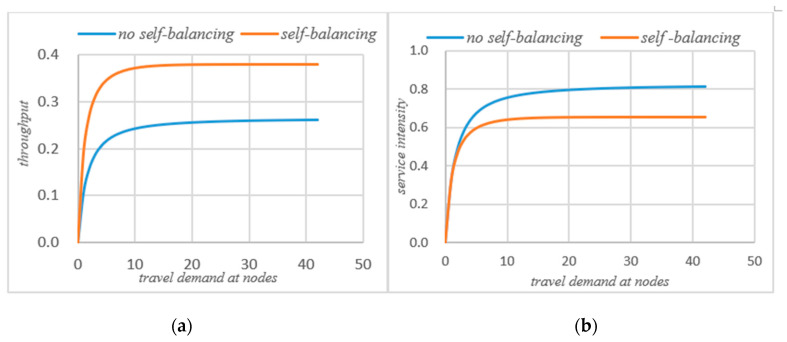
(**a**) System throughput comparison; (**b**) system service intensity comparison.

As can be seen that after self-balancing, the actual throughput of node 1 has decreased, while the throughput of nodes 2 and 3 has increased significantly. According to Figure 8a, after the self-balancing of autonomous vehicles, the average throughput of nodes have increased significantly. Figure 6a,b compare the changes of service intensity at the nodes before and after the self-balancing of autonomous vehicles in the system under the same travel conditions. The horizontal axis represents travel demand and the vertical axis represents service intensity.

Figure 6 shows that on the basis of meeting the traffic demand of the system, the service intensity of node 1 is slightly increased after self-balancing, but the service intensity of node 2 and node 3 is significantly reduced. According to Figure 8b, if the system’s autonomous vehicles adopt the self-balancing measures, the average service intensity at nodes will be reduced on the basis of meeting the traffic demand.

### 4.2. Comparative Analysis before and after System Self-Balancing

The purpose of the autonomous vehicles self-balancing strategy studied in this paper is to optimize the traffic operation state of the entire road network, not for the status of a single node in the road network, so in the process of self-balancing optimization, there may be some situations, such as a slight increase in the queue length of a node and so on. However, the self-balancing process of autonomous vehicles improves the overall operating efficiency of the road network, saves the waiting time of the system, increases the actual throughput of the system, and reduces the service intensity of the system on the basis of meeting the travel requirements of the system. Therefore, the self-balancing system has an indelible effect on the transportation network of fully autonomous vehicles, as shown in Figure 7 and Figure 8 below.

It can be seen from Figure 7 that if the system adopts the strategy of self-balancing, the average waiting time of customers in the system will be significantly reduced. Figure 8 shows the change of average queue length before and after self-balancing. Since the system is a closed-loop queuing network, the total number of customers remains unchanged, so no matter how to optimize the travel index of the system, the total average queue length of the system remains unchanged.

Figure 8a shows the change of the actual throughput of the system before and after self-balancing. Figure 8b shows the change of average service intensity before and after self-balancing. It could be seen that after self-balancing, the throughput of the system increases significantly, while the service intensity decreases.

In summary, adopting the self-balancing strategy for the road network of fully autonomous vehicles can not only increase the actual number of trips serviced by the road network but also effectively alleviate the service intensity of the road network. On the basis of ensuring customers’ trips, it effectively reduces the system’s average waiting time as well as balances the queue length and service intensity at nodes.

## 5. Conclusions

The article projects the defined self-balancing traffic system of autonomous vehicles into the Jackson closed-loop network, makes full use of the intelligent characteristics of the self-driving vehicles, and then establishes a self-balancing system model based on the fixed number of customers, so as to balance the utilization rate of the autonomous vehicles in the system through the potential travel demand and avoid the difficulty of parking private cars in the current transportation system, as well as the unbalanced allocation of vehicle resources in the road network caused by automatic parking during holidays or morning and evening peaks, which results in an uneven distribution of vehicle resources and low vehicle service efficiency phenomenon in the road network. Through the MVA method, the calculation difficulty of the system performance index is reduced, and a case analysis is carried out in the fourth part. The results show that the model could well balance the allocation of vehicle resources in the system and significantly reduce the average waiting time of system customers. While ensuring demand, it could not only increase the travel service of the system but also significantly reduce the service intensity of nodes and relieve the service pressure of the system. It could fundamentally solve the phenomenon of concentrated idleness of vehicles in the current traffic after use, maximize the use of mobile vehicles in the system, and realize the self-balance of the traffic network. Of course, there are still some imperfections in this paper. In the future, we could consider whether the increase in the number of service trips in the self-balancing process of autonomous driving can offset the increase in operating costs during the self-balancing process or consider the phenomenon of carpooling to earn more benefits.

## Data Availability

The study did not report any data.
